# Metformin Prevents Nonunion after Three-Cannulated-Screw Fixation in Displaced Femoral Neck Fractures: A Retrospective Study

**DOI:** 10.1155/2016/5682541

**Published:** 2016-11-20

**Authors:** Xiao-zhong Zhu, Xian-you Zheng

**Affiliations:** Department of Orthopaedic Surgery, Shanghai Jiaotong University Affiliated Sixth People's Hospital, Shanghai 200233, China

## Abstract

Patients aged from 40 to 60 with displaced fractures of the femoral neck (Garden III or IV) who received fixation with three cannulated screws from January 2005 to December 2012 were evaluated retrospectively for the development of nonunion. Plasma HbA1C, a marker for long-term plasma glucose level, anti-T2DM medication, and other potential risk factors were recorded for the purpose of this study. There were no differences between the union and nonunion groups with respect to age, gender, Garden classification, Pauwel's angle, reduction quality, and T2DM presence. There were significant differences in reduction quality and preoperative plasma HbA1C level between patients with and those without union. The odds ratio (OR) for fracture nonunion was 2.659 (95% confidence interval [CI], 1.530–4.620) in subjects with anatomical reduction compared with those without anatomical reduction, 4.797 (95% CI, 1.371–16.778), in subjects with poor blood glucose control (HbA1C > 10%). The metformin usage showed a preventive effect on nonunion development (OR: 0.193 and CI: 0.060–0.616). The nonunion rate of metformin group (6.7%, 6/89) was even much lower than that of patients without T2DM (17.4%, 80/460).

## 1. Background

The nonunion is one of the common complications after femoral neck fractures. Reported incidence of nonunion after three-cannulated-screw fixation ranged from 13.3% to 21.8% and many risk factors including impropriate crew fixation configuration, displaced fracture, and poor reduction have been revealed [1–3].

Type 2 diabetes mellitus (T2DM) affects 382 million individuals across the world and its prevalence is steadily rising [4]. T2DM leads to the fragility of bones by affecting the bone metabolism [5]. Emerging evidences have indicated T2DM as an independent risk factor for fractures [6, 7]. Hence, management of fractures in T2DM patients has become an increasingly important topic in orthopedic practice and research.

T2DM is characterized by a series of micro- and macrovascular complications caused by insulin resistance [8]. In addition, insulin resistance restrains the reestablishment of vascularization, thereby impairing the wound healing [9, 10]. Previous study has demonstrated that T2DM is associated with impaired fracture healing including delayed union and nonunion [11]. The nonunion after foot and ankle surgery is more common in patients with T2DM [12]. However, the influence of T2DM on femoral neck nonunion is not clear.

## 2. Methods

All patients with fresh displaced femoral neck fractures (Garden III or IV) who received surgical fixation with three cannulated screws from January 2005 to December 2012 in our hospital were assessed for eligibility for study inclusion. The following inclusion criteria were applied: age from 40 to 60, surgery time within 30 days after the injury, and having glycated hemoglobin (HbA1C) detection before surgery. Patients with any of the following criteria were excluded: T1DM, pathological fracture, additional fracture in the ipsilateral lower limb or pelvis, and loss of follow-up within 24 months after surgery. A total of 601 patients were included in our analysis. Data from the included patients were analyzed retrospectively. The following preoperative variables were recorded: age, gender, body mass index (BMI), and glycated hemoglobin (HbA1C). Demographic information and characteristics of the fractures before surgery was summarized in Table 1. T2DM-related medication after surgery was recorded before analysis. The T2DM-related medications for each patient were summarized in Supplementary Table 1, in Supplementary Material available online at http://dx.doi.org/10.1155/2016/5682541. According to previous studies, nonunion was defined as a loss of reduction or fixation after six weeks or radiological absence of union at one year [1, 13].

Statistical analysis was performed using SPSS 22.0 software. A *P* value of < 0.05 was considered to be statistically significant. Fisher's exact test was used to compare nonunion rates between the groups. Data were presented as mean ± standard deviation.

## 3. Results

All the potential risk factors, including Garden classification, Pauwel's angle, reduction quality, T2DM presence, preoperative plasma HbA1C level, and T2DM-related medications, were considered as covariates, respectively. The development of nonunion was considered as dependent variable. The results of multivariate binary logistic regression analysis were shown in Table 2. Reduction quality instead of the fractures characteristics was associated with the occurrence of nonunion, which is consistent with previous studies [14]. The presence of T2DM did not significantly increase or decrease the nonunion rate in this study. The OR for fracture nonunion was 4.797 (95% CI, 1.371–16.778) in subjects with poor blood glucose control (HbA1C > 10%).

We also analyzed whether T2DM-related medications were associated with the nonunion rate. The metformin usage showed a preventive effect on nonunion development (OR: 0.183 and CI: 0.058–0.577) while other T2DM-related medications showed no such effect. There was no significant difference in preoperative plasma HbA1C level between T2DM patients with and without metformin usage (*P* > 0.05, Student's *t*-test) suggesting the prevention of metformin might be irrelevant to the anti-T2DM effect and improved T2DM control. Supporting our notion, the nonunion rate of metformin group (6.7%, 6/89) was even much lower than that of patients without T2DM (17.4%, 80/460).

## 4. Discussion

In this study, we found that the T2DM presence was not an independent risk factor for fracture nonunion. Increased risks for the development of fracture nonunion after internal fixation of femoral neck fractures were only found in patients with poor blood glucose control. Our observation suggested that the inhibitory effect of T2DM on fracture healing might be negated after ideal control of plasma glucose level.

Metformin has been shown to have osteogenesis-promoting effects in vitro [15]. In addition, metformin inhibits osteoclast differentiation by reducing receptor activator of nuclear factor *κ*B ligand expression [16]. Theoretically, metformin could promote bone formation and thus facilitate fracture healing. However, the studies based on animal models have shown controversial effects of metformin on fracture healing [17, 18]. Our study supported the notion that metformin might prevent the nonunion after femoral neck fracture by providing clinical evidences.

It is possible that the preventive effect of metformin was associated with its glucose-lowering effect. The preoperative plasma HbA1C levels between T2DM patients with and those without metformin usage had no significant difference suggesting the preventive effect of metformin might be independent of plasma glucose control. In addition, other anti-T2DM medications showed no preventive effect on nonunion. Most importantly, the nonunion rate of metformin group was even significantly lower compared to patients without T2DM.

Since the side effects of metformin are mild, patients with femoral fractures might benefit from usage of metformin even without T2DM presence. Future perspective studies and clinical trials might further confirm our observation.

We also found that fracture without anatomical reduction was a significant risk factor for nonunion in displaced femoral neck fractures, whereas fracture angle was not. A more horizontal fracture line was associated with internal fixation failure in clinical and mechanical studies [19]. However, we did not find correlation between Pauwel's classification and the development of nonunion.

This study has some limitations. The T2DM-related medications were recorded before analysis by contacting participants and thereby the recall bias could not be entirely avoided. However, the T2DM-related medication is generally applied in long term and the alteration on antidiabetic drugs in T2DM patients is uncommon. Hence, the recall bias might be mild and main conclusion of this study is not affected. Clearly, a perspective study is required for further confirmation on our findings.

## 5. Conclusion

Metformin can decrease the nonunion rate of femoral neck fractures independent of its anti-T2DM effect.

## Supplementary Material

Metformin, insulin, alpha-glucosidase inhibitor and secretagogue were most widely in treatment of T2DM. The usage of these therapies in each patient was therefore recorded and summarized.

## Figures and Tables

**Table 1:** Demographic information and characteristics of the fractures.

	Total (*n* = 601)	Union (*n* = 503)	Nonunion (*n* = 98)	*P* value
Male	337	290	47	0.077
Female	264	213	51	
Age^†^	50.2 ± 5.1	50.2 ± 5.2	49.8 ± 5.1	0.471
BMI^†^	23.1 ± 2.4	23.1 ± 2.4	22.9 ± 2.2	0.428
Garden classification^*∗*^				
Stage III	350	298	52	0.257
Stage IV	251	205	46	
Pauwel's classification^*∗*^				
Stage I	55	44	11	0.493
Stage II	368	313	55	
Stage III	178	146	32	
Reduction quality^*∗*^				
Anatomical	519	446	73	0.0002
Nonanatomical	82	57	25	
T2DM^*∗*^				
−	460	380	80	0.193
+	141	123	18	
HbA1C^§^				
<6%	475	393	82	0.041
6–10%	100	91	9	
>10%	26	19	7	
Metformin^*∗*^				
−	512	420	92	0.008
+	89	83	6	
Insulin^*∗*^				
−	526	435	91	0.081
+	75	68	7	
Alpha-glucosidase inhibitor^§^				
−	573	477	96	0.179
+	28	26	2	
Secretagogue^*∗*^				
−	549	458	91	0.561
+	52	45	7	

^*∗*^ *P* values are based on the chi-square test. ^†^ *P* values are based on Student's *t*-test. ^§^ *P* values are based on the Fisher exact test.

**Table 2:** Results of multivariate binary logistic regression analysis.

	*P* value	OR	95% CI
Garden Stage IV (reference: Garden Stage III)	0.284	1.280	0.814–2.013
Pauwel's Stage II (reference: Pauwel's Stage I)	0.223	0.630	0.300–1.323
Pauwel's Stage III (reference: Pauwel's Stage I)	0.443	1.219	0.735–2.022
Reduction quality (reference: anatomical)^*∗*^	0.001	2.659	1.530–4.620
T2DM (+)	0.241	3.030	0.474–19.356
HbA1C 6%–10% (reference: HbA1C < 6%)	0.385	2.341	0.343–15.984
HbA1C > 10% (reference: HbA1C < 6%)^*∗*^	0.014	4.797	1.371–16.778
Metformin (+)^*∗*^	0.005	0.193	0.060–0.616
Insulin (+)	0.101	0.386	0.124–1.204
Alpha-glucosidase inhibitor (+)	0.363	0.459	0.086–2.456
Secretagogue (+)	0.875	1.094	0.356–3.362

OR = odds ratio; CI = confidence interval. ^*∗*^ *P* < 0.05.

## Abbreviations

T2DM: Type 2 diabetes mellitus
OR: Odds ratio
CI: Confidence interval
HbA1C: Hemoglobin A1C.

## Additional Points

*Availability of Data and Materials*. The dataset supporting the conclusions presented in this article is available on request from the corresponding author.
